# Mechanisms of accidental fall injuries and involved injury factors: a registry-based study

**DOI:** 10.1186/s40621-020-0234-7

**Published:** 2020-03-16

**Authors:** Tatiana Nikolaevna Unguryanu, Andrej Mechislavovich Grjibovski, Tordis Agnete Trovik, Børge Ytterstad, Alexander Valerievich Kudryavtsev

**Affiliations:** 1 Department of Community Medicine, UiT - The Arctic University of Norway, Hansine Hansens veg 18, Tromsø, Norway; 2 Arkhangelsk International School of Public Health, Northern State Medical University, Troitsky Ave., 51, Arkhangelsk, 163000 Russia; 3 North-Eastern Federal University, Belinsky str., 58, Yakutsk, 677027 Russia; 4 Al-Farabi Kazakh National University, Al-Farabi Ave., 71, Almaty, Kazakhstan 050040; 5 West Kazakhstan Marat Ospanov State Medical University, Maresyev str., 68, Aktobe, 030019 Kazakhstan

**Keywords:** Fall injuries, Injury registry, Shenkursk, Cluster analysis

## Abstract

**Background:**

Falls are the leading cause of injury-related morbidity and mortality worldwide, but fall injury circumstances differ by age. We studied the circumstances of accidental fall injuries by age in Shenkursk District, Northwest Russia, using the data from the population-based Shenkursk Injury Registry.

**Methods:**

Data on accidental fall injuries (hereafter: fall injuries) occurring in January 2015–June 2018 were extracted from the Shenkursk Injury Registry (*N* = 1551) and categorized by age group (0–6, 7–17, 18–59, and 60+ years). The chi-square test and ANOVA were used to compare descriptive injury variables across age groups, and a two-step cluster analysis was performed to identify homogeneous groups of fall injuries by preceding circumstances.

**Results:**

Half of recorded fall injuries in the 0–6 year age group occurred inside dwellings (49%). The largest cluster of falls (64%) mainly included climbing up or down on home furnishings. In the 7–17 year age group, public outdoor residential areas were the most common fall injury site (29%), and the largest cluster of falls (37%) involved physical exercise and sport or play equipment. Homestead lands or areas near a dwelling were the most typical fall injury sites in the age groups 18–59 and 60+ years (31 and 33%, respectively). Most frequently, fall injury circumstances in these groups involved slipping on ice-covered surfaces (32% in 18–59 years, 37% in 60+ years).

**Conclusion:**

The circumstances of fall injuries in the Shenkursk District varied across age groups. This knowledge can be used to guide age-specific preventive strategies in the study area and similar settings.

## Background

Falls follow road traffic accidents as the second leading cause of injury-related mortality worldwide (World Health Organization 2014). Based on the latest Global Health Estimates, 660,320 people died from falls in 2016 in the world (World Health Organization 2018). The proportion of fall injury-related mortality in the World Health Organization European Region increased from 12% in 2000 to 17% in 2016 (World Health Organization 2018; World Health Organization n.d.).

Children and older people are highly prone to falls. Among children, fall injury-related mortality is rare, but rates of hospitalization and visits to the emergency department are high (Lee et al. 2017; Ali et al. 2019), with falls accounting for 25–52% of hospitalizations among children in developing countries (Kaida et al. 2006). For the elderly, falls are the most common cause of injury-related mortality, the rates of which increase exponentially with age (Yoshida 2007).

Fall injuries occur because of endogenous and environmental factors. Previous studies have described demographic, social, medical, and temporal characteristics of fall injuries (Lee et al. 2017; Kaida et al. 2006; Peel et al. 2002; Stevens and Sogolow 2005). Some studies have examined the environmental and behavioral circumstances of fall injuries by age group: in children (Ali et al. 2019; Istre et al. 2003), young adults, middle-aged adults (Timsina et al. 2017; Dandona et al. 2010), and elderly people (Orces and Alamgir 2014). Fall injuries in preschool-aged children most often occur at home, and the most serious injuries are caused by falls on stairs, from furniture, and from playground equipment (Pickett et al. 2003; Flavin et al. 2006; Chaudhary et al. 2018; Wadhwaniya et al. 2017). The incidence of home injuries among children decreases with age. In school-aged children, fall injuries are more common in school environments, public parks, on highways or roads, and in sports/recreational facilities (Unni et al. 2012). Young and middle-aged adults typically fall outdoors, while and the elderly most commonly fall at home (Talbot et al. 2005). Knowledge of the typical site-specific and group-specific upstream circumstances of fall injuries is required for effective prevention. Therefore, local data on where, when, and how typical fall circumstances occur are necessary to create preventive strategies at a country or community level (Ytterstad 1996).

The Shenkursk Injury Registry (SHIR) is a population-based registry that was established in the Shenkursk District, Northwestern Russia in 2015 for the purposes of primary prevention (Unguryanu et al. 2019), and it shows that falls are the most common injury mechanism in the area. Using data from the SHIR, we studied the circumstances of accidental fall injuries (hereafter referred to as fall injuries) by age to provide data-driven evidence for development of preventive strategies.

## Methods

### Study site

The population of Shenkursk District was 12,610 on 1 January 2018. The area has a cold climate with a mean annual air temperature of 1.4 °C, and temperatures below zero prevail from October to April (Climate: Shenkursk n.d.). The economy of the district is based on forestry, woodworking, and agriculture. The road network is grossly underdeveloped, and most local roads are unpaved. Health care in the district is largely provided by the central district hospital (CDH), which offers in-patient facilities (62 beds) and out-patient polyclinics for adults and children.

### The Shenkursk injury registry and selection criteria

The SHIR records all injuries (ICD-10 diagnoses from S00 to T78) treated at the Shenkursk CDH. Data are collected using a universal injury registration form (IRF) - a two-page sheet with sections for recording patients’ socio-demographic characteristics (sex, date of birth, address of residence, place of work or study), information about time and place of the injury, alcohol consumption in the 24 h before injury, use of protective equipment, and optional sections for descriptions of road traffic and sport injuries. The IRF also has a field for a free-text description of how the injury has occurred. This field is supplemented by three supportive questions to facilitate detailed descriptions of injury circumstances: “What were you doing?”, “What went wrong?”, and “How were you injured?”. These free-text descriptions are transformed into several upstream categorical variables using the corresponding coding lists (i.e., mechanism of preceding activity, accident mechanism, and injury mechanism) and factors involved (i.e., factors involved in mechanism of preceding activity, factors involved in accident mechanism, and factors involved in injury mechanism). The concluding part of the IRF has several fields to be completed by a physician, including the diagnosis with corresponding ICD-10 code, injury severity according to the Abbreviated Injury Scale (AIS), and whether the patient was hospitalized. Physicians who provide treatment for injuries at the Shenkursk CDH or in its ambulance cars are instructed to offer the IRF to each treated patient at their first outpatient or ambulance visit, or within few days after hospitalization. Patients complete IRFs, often with the assistance of accompanying relatives, a nurse, or a physician. If a patient does not complete the IRF due to a severe condition or other reasons (about 40% of cases), injury registrars (two trained nurses) complete the form retrospectively, using data from routine medical records (ambulance journal, outpatient medical card, case history) as well as information obtained from the attending physician. A more detailed description of the SHIR, the IRF, and methodological considerations are presented elsewhere (Unguryanu et al. 2019; Unguryanu et al. 2017).

In the present analysis, we used data on nonfatal fall injuries that occurred from January 2015 to June 2018 and were registered in the SHIR. A fall injury was defined as “inadvertently coming to rest on the ground, floor or other lower level” (ICD-10 code W00–19) (Istre et al. 2003).

### Data analysis

Fall injuries were categorized into four age groups: preschool age (0–6 years), school age (7–17 years), working age (18–59 years), and elderly age (60+ years). Categorical injury characteristics are presented as absolute numbers and percentages. The AIS is presented as mean (standard deviation). Chi-square test and one-way ANOVA were used when comparing fall injury subgroups.

A two-step cluster analysis was performed to identify homogeneous groups of fall injuries by injury circumstances for each of the four age groups. Five upstream variables originating from free-text descriptions of injury circumstances were included in cluster analyses: mechanism of preceding activity, accident mechanism, injury mechanism, factors involved in mechanism of preceding activity, and factors involved in accident mechanism. The number of clusters was determined automatically using the Bayesian information criterion. The average silhouette measure of cohesion and separation (range: − 1 to + 1) was used to indicate the overall goodness of fit (Rousseeuw 1987). It is conventionally accepted that a silhouette measure of < 0.2 is considered poor, between 0.2 and 0.5 indicates a fair solution, and > 0.5 is a good solution (Mooi and Sarstedt 2011). All statistical analyses were performed using SPSS, version 25 (SPSS Inc., Chicago, IL, USA).

## Results

Altogether, the SHIR contained data on 1551 fall injuries occurring during the study period (Table 1), which constituted 38% of all registered injuries in the period. There were 84 fall injuries (5.4%) in the preschool age group, 294 (18.9%) in the school age group, 734 (47.3%) in the working age group, and 437 (28.2%) in the elderly age group. The distribution of fall injuries by injury site, injury localization, injury severity, proportion of hospitalizations, and proportion of injuries that occurred in the cold season (15 October-14 April) were significantly different across age groups.

**Table 1:** Demographic, medical, and temporal characteristics of accidental fall injuries, Shenkursk District, January 2015–June 2018

	Age groups**			
	Preschool 0–6 years (*N* = 84)	School 7–17 years (*N* = 294)	Working 18–59 years (*N* = 736)	Elderly 60+ years (*N* = 437)
	n (%)	n (%)	n (%)	n (%)
Sex, male	44 (52.4)	168 (57.1)	377 (51.2)	140 (32.0)
Injury site				
Homestead land, area near a dwelling	19 (22.6)	34 (11.6)	227 (30.8)	144 (33.0)
Dwelling, inside parts (living room, bedroom, kitchen)	41 (48.8)	27 (9.2)	119 (16.2)	121 (27.7)
Dwelling, outer parts (roof, porch)	2 (2.4)	12 (4.1)	86 (11.7)	36 (8.2)
Roadway	4 (4.8)	20 (6.8)	73 (9.9)	52 (11.9)
Public outdoor residential area	7 (8.3)	85 (28.9)	133 (18.1)	56 (12.8)
Educational institution (inside and outside, excl. Sports facilities)	2 (2.4)	37 (12.6)	3 (0.4)	1 (0.2)
Sports facilities and playgrounds	8 (9.5)	68 (23.1)	21 (2.9)	—
Natural surroundings (forest, field, river side)	1 (1.2)	3 (1.0)	45 (6.1)	14 (3.2)
Other*	—	8 (2.8)	29 (3.9)	13 (3.0)
Injury localization, ICD-10				
S00–09: Head	27 (32.1)	36 (12.2)	83 (11.3)	31 (7.1)
S20–29: Thorax	5 (6.0)	9 (3.1)	120 (16.3)	72 (16.5)
S30–39: Abdomen, lower back, lumbar spine and pelvis	—	18 (6.1)	45 (6.1)	19 (4.3)
S40–69: Upper extremity	35 (41.7)	122 (41.4)	260 (35.3)	180 (41.2)
S70–99: Lower extremity	17 (20.2)	103 (35.0)	219 (29.8)	127 (29.1)
Other*	—	6 (2.1)	9 (1.3)	8 (1.9)
Injury severity according to the AIS				
1 Minor	55 (65.5)	207 (70.4)	421 (57.2)	190 (43.5)
2 Moderate	20 (23.8)	80 (27.2)	234 (31.8)	162 (37.1)
3 Severe, but not life-threatening	9 (10.7)	6 (2.0)	79 (10.7)	83 (19.0)
4 Severe, potentially life-threatening and critical, with uncertain survival	—	1 (0.3)	2 (0.2)	2 (0.5)
Hospitalization, yes	11 (13.1)	19 (6.5)	89 (12.1)	88 (20.1)
Season				
15 October - 14 April	24 (28.6)	177 (59.9)	483 (65.5)	266 (60.4)
15 April - 14 October	60 (71.4)	117 (40.1)	253 (34.5)	171 (39.6)
Alcohol consumption in preceding 24 h, yes	–	3 (1.0)	136 (18.5)	22 (5.0)

*ICD* International Classification of Diseases, *AIS* Abbreviated Injury Scale

*Combines categories accounting for < 5% of observations in all age groups

**p-values for group comparisons on all presented characteristics are < 0.001

Mechanism of preceding activity, accident mechanism, injury mechanism, and factors involved in the mechanism of preceding activity, accident mechanism, and injury mechanism also differed by age group (Table 2).

**Table 2:** Mechanisms of accidental fall injuries and involved factors, Shenkursk District, January 2015–June 2018

	Age groups**			
	Preschool 0–6 years (*N* = 84)	School 7–17 years (*N* = 294)	Working 18–59 years (*N* = 736)	Elderly 60+ years (*N* = 437)
	n (%)	n (%)	n (%)	n (%)
Mechanism of preceding activity				
Walking	14 (16.7)	111 (37.8)	414 (56.3)	269 (61.6)
Physical exercise	16 (19.0)	79 (26.9)	31 (4.2)	4 (0.9)
Running	13 (15.5)	34 (11.6)	4 (0.5)	—
Climbing up/down	18 (21.4)	18 (6.1)	14 (1.9)	5 (1.1)
Going on stairs	6 (7.1)	29 (9.9)	70 (9.5)	34 (7.8)
Sitting and lying	13 (15.5)	6 (2.0)	14 (1.9)	23 (5.3)
Standing	4 (4.8)	9 (3.1)	59 (8.0)	25 (5.7)
Working at home or garden	—	6 (2.0)	64 (8.7)	29 (6.6)
Carrying something	—	2 (0.7)	66 (9.0)	48 (11.0)
Factors involved in mechanism of preceding activity				
No factor reported	26 (31.0)	150 (51.0)	382 (51.9)	267 (61.1)
Sport and play equipment	13 (15.5)	80 (27.2)	29 (3.9)	4 (0.9)
Stairs in a building	5 (6.0)	24 (8.2)	34 (4.6)	8 (1.8)
Furniture	17 (20.2)	3 (1.0)	3 (0.4)	12 (2.7)
Wooden object	4 (4.8)	4 (1.4)	54 (7.3)	15 (3.4)
Porch	1 (1.2)	6 (2.0)	59 (8.0)	30 (6.9)
Loose household items	—	—	34 (4.6)	34 (7.8)
Other factors*	18 (21.5)	27 (9.2)	141 (19.1)	67 (15.3)
Accident mechanism				
Slipping	13 (15.5)	101 (34.4)	426 (57.9)	216 (49.4)
Stepping wrong	14 (16.7)	68 (23.1)	122 (16.6)	51 (11.7)
Stumbling over something	13 (15.5)	41 (13.9)	74 (10.1)	76 (17.4)
Loss of balance	35 (41.7)	55 (18.7)	47 (6.4)	17 (3.9)
Faintness	—	—	14 (1.9)	62 (14.2)
Other*	9 (10.8)	29 (9.9)	53 (7.2)	15 (3.5)
Factors involved in accident mechanism				
Internal human factor	46 (54.8)	114 (38.8)	161 (21.9)	124 (28.4)
Ice-covered surface	6 (7.1)	85 (28.9)	337 (45.8)	173 (39.6)
Sport and play equipment	6 (7.1)	23 (7.8)	11 (1.5)	3 (0.7)
Another human	6 (7.1)	14 (4.8)	9 (1.2)	—
Part of building inside	2 (2.4)	12 (4.1)	22 (3.0)	26 (5.9)
Wooden object	3 (3.6)	9 (3.1)	39 (5.3)	26 (5.9)
Wet surface outside / inside	1 (1.2)	10 (3.4)	62 (8.4)	35 (8.0)
Furniture	6 (7.1)	2 (0.7)	15 (2.0)	3 (0.7)
Other factors*	8 (9.5)	25 (8.5)	80 (10.8)	47 (10.7)
Injury mechanism				
Fall on the same level	37 (44.0)	220 (74.8)	520 (70.7)	360 (82.4)
Fall on stairs	10 (11.9)	37 (12.6)	138 (18.8)	56 (12.8)
Fall from a height of <1.5 m	32 (38.1)	17 (5.8)	33 (4.5)	17 (3.9)
Fall from a height of > 1.5 m	5 (6.0)	20 (6.8)	45 (6.1)	4 (0.9)
Factors involved in injury mechanism				
Surface outside	31 (36.9)	183 (62.2)	489 (66.4)	245 (56.1)
Surface inside	31 (36.9)	67 (22.8)	119 (16.2)	109 (24.9)
Wooden object	3 (3.6)	13 (4.4)	52 (7.1)	34 (7.8)
Sport and play equipment	—	16 (5.4)	5 (0.7)	—
Furniture	11 (13.1)	1 (0.3)	15 (2.0)	23 (5.3)
Other factors*	8 (9.5)	14 (4.8)	56 (7.6)	26 (5.9)

*Combines categories accounting for < 5% of observations in all age groups

** p-values for group comparisons on all presented characteristics are < 0.001

Among the preschool age group, the most common fall injury site was inside of dwellings (49%). For the school age group, the most common fall injury site was public outdoor residential areas (29%), while the most common fall injury site for individuals in the working age group and elderly age group was homestead land or area near a dwelling (31 and 33% respectively). Upper and lower extremities were the most commonly injured body parts in all age groups.

According to the AIS, minor injuries constituted about two-thirds of fall injuries in the preschool age, school age, and working age groups (57–70%); and they constituted almost half of fall injuries in the elderly age group (44%). The proportion of hospitalizations was the highest in the elderly age group (20%). The proportion of fall injuries in the cold season in the preschool age group (29%) was a half of that in other age groups. About 19% of fall injuries in the working age group had a report of alcohol consumption in the preceding 24 h.

The most common mechanism of preceding activity in the working and elderly age groups was walking (56 and 62%, respectively), in the preschool age group it was climbing up/down (21%), and in the school age group it was walking (38%) and physical exercise (27%) (Table 2). Slipping was the most frequent accident mechanism in the working (58%) and elderly (49%) age groups. Injury mechanisms showed that most falls were at the same level in all age groups, but the preschool age group had a relatively higher proportion (38%) of fall injuries from a height of < 1.5 m. Most cases in all age groups reported no factors involved in the mechanism of preceding activity. An internal human factor (e.g., loss of balance, dizziness, weakness) was the most commonly mentioned factor involved in accident mechanism in the preschool (55%) and school (39%) age groups. In working and elderly age groups, the most common factor involved in accident mechanism was ice-covered surfaces (46 and 40% of cases, respectively). Surfaces outside were the most frequent category of factors involved in injury mechanism in all age groups except preschool (ranging between 56 and 62%). For the preschool age group, the proportions of surfaces inside and outside that acted as factors involved in injury mechanism were similar (37%).

**Fig. 1:**
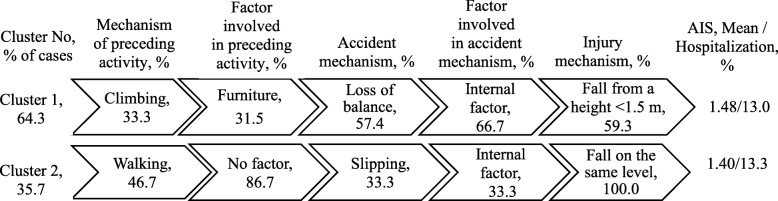
Clusters of fall injuries, children 0–6 years, Shenkursk District, January 2015–June 2018 (*N* = 84). Average silhouette = 0.4. AIS: Abbreviated Injury Scale

The fewest fall injuries were recorded in the preschool age group, which formed two clusters (Fig. 1). Cluster 1 (64% of cases) mainly included fall injuries involving climbing up or down (on furniture, play equipment, stairs) and a loss of balance. Cluster 2 (36% of cases) included falls on the same level, most commonly preceded by walking and slipping.

**Fig. 2:**
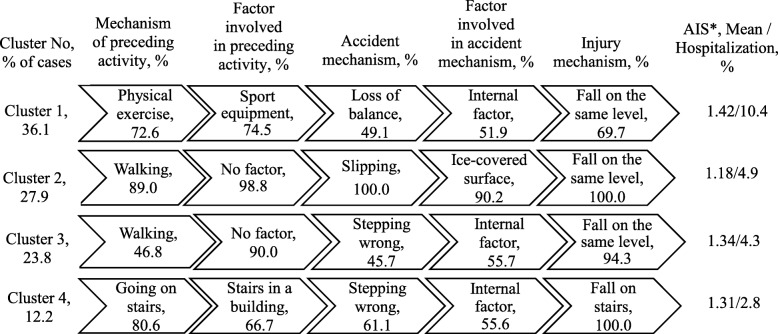
Clusters of fall injuries, children 7–17 years, Shenkursk District, January 2015–June 2018 (*N* = 294). Average silhouette = 0.5. * *F* = 3,32; d.f. = 3, *p* = 0.002 between clusters. AIS: Abbreviated Injury Scale

Four clusters of fall injuries were identified in the school age group (Fig. 2). Cluster 1 (36% of cases) largely involved cases which occurred during physical exercise and involved sport or play equipment, and a loss of balance. Cluster 2 (28%) largely constituted fall injuries that resulted from slipping on ice-covered surfaces while walking. Cluster 3 (24%) mainly consisted of cases who walked and fell due to stepping wrong. Fall injuries involving stairs in buildings - walking up or down and falling due to stepping wrong - comprised cluster 4 (12%).

**Fig. 3:**
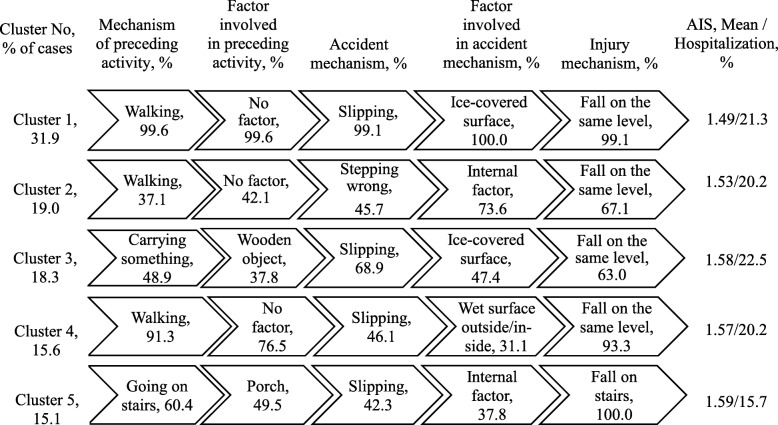
Clusters of fall injuries, adults 18–59 years, Shenkursk District, January 2015–June 2018 (*N* = 736). Average silhouette = 0.4. AIS: Abbreviated Injury Scale

Analyses of the fall injuries in the working age group resulted in five clusters (Fig. 3). For cluster 1 (32% of cases) and cluster 3 (18% of cases), the most common accident mechanism was slipping, with an ice-covered surface being the most common involved factor. The difference between them was in the mechanism of preceding activity: simple walking preceded the fall in almost all cases in cluster 1, while in cluster 3 the mechanism was carrying something. Cluster 2 (19% of cases) mainly constituted fall injuries while walking, which occurred due to stepping wrong. Cluster 4 (16% of cases) was largely constituted by fall injuries resulting from slipping on a wet surface outside or inside. Cluster 5 (15%) was entirely made up of falls on stairs.

**Fig. 4:**
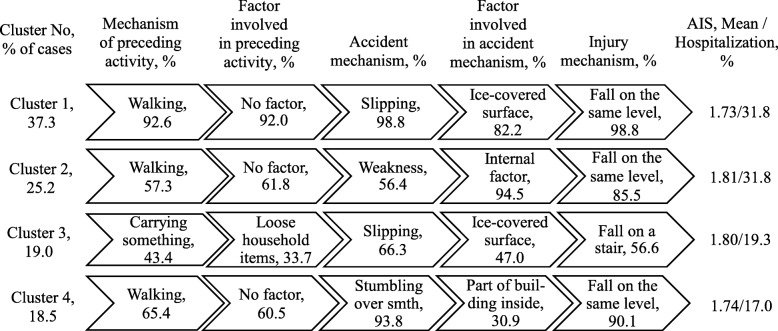
Clusters of fall injuries, adults 60+ years, Shenkursk District, January 2015–June 2018 (*N* = 437). Average silhouette = 0.5. AIS: Abbreviated Injury Scale

Four clusters of fall injuries were identified in the elderly age group (Fig. 4). For cluster 1 (37% of cases) and cluster 3 (19% of cases), the situation was similar to that in the working age group. Cluster 2 (25% of cases) contained cases in which fall injuries mainly occurred due to faintness while walking. Cluster 4 (18% of cases) consisted largely of fall injuries during walking, where stumbling over something was the key accident mechanism.

Mean AIS values were significantly different between the clusters of fall injuries only in the school age group (р = 0.002) (Fig. 2). The highest mean AIS value was observed in Cluster 1, which accumulated fall injuries during exercising. Correspondingly, fall injuries in cluster 1 had the highest proportion of hospitalization (10%), but the difference between the clusters was not significant.

## Discussion

In the preschool age group, fall injuries were most commonly associated with climbing on to or down from home furnishings. The most substantial part of fall injuries among the school age group occurred during physical exercise involving sport or play equipment. The most frequent accident mechanism in the working and elderly age groups was slipping on an ice-covered surface.

Our findings in preschool children are similar to observations from Bangladesh (Wadhwaniya et al. 2017) and Canada (Flavin et al. 2006), which showed that the majority of injuries in the age group 0–6 years occurred at home. In our study, the most common factor involved in mechanism of preceding activity in the preschool age group was furniture, which is in line with findings from other studies (Ali et al. 2019; Pickett et al. 2003; Chaudhary et al. 2018; Unni et al. 2012; Savitsky et al. 2007; Pitone and Attia 2006).

The proportion of head injuries in the preschool age group (32%) was three times higher than that among the school age group, and the proportions of severe injuries (11%) and hospitalization (13%) were five and two times higher, respectively (Park et al. 2004). These findings are supported by studies showing that head injuries are common among the youngest children (Ali et al. 2019; Pickett et al. 2003; Flavin et al. 2006; Wadhwaniya et al. 2017; Pitone and Attia 2006), which may be explained by a limited child’s ability to shield the head during a fall (Park et al. 2004). Based on our findings, prevention of fall injuries in preschool children should primarily address the safety of the home environment. It is vitally important to prevent preschool children from climbing high up without parental supervision and to cover floors with soft materials near places where children can climb up on furniture or other objects in the home.

Our study showed that the school age group most commonly sustained fall injuries outside during sport and play activities, and this is comparable to findings in other settings (Ali et al. 2019; Wadhwaniya et al. 2017; Unni et al. 2012; Gupta et al. 2016; Schneuer et al. 2018). Therefore, fall injury prevention in school children should focus on safe sports and play, by promoting the use of helmets, protective padding, and safety nets. Safety education efforts should also include school teachers.

Slipping on ice-covered surfaces was the most frequent fall injury in the working and elderly age groups, which is in concordance with studies carried out in Norway, Finland, the UK, the USA, and Iran (Bulajic-Kopjar 2006; Flinkkilä et al. 2011; Mardani-Kivi et al. 2014; Gevitz et al. 2017; Ralis 1981). These findings suggest that preventive measures in Shenkursk District and settings with similar climatic conditions should primarily target removing ice and preventing slipping on icy surfaces, for example, by spreading sand and wearing shoes with high-friction outsoles or spikes.

It is also worth noting that alcohol consumption in the preceding 24 h was reported in connection to one-fifth of fall injuries in the working age group, and this may be underreported. Due to our concern about the latter, alcohol was not considered as a factor in cluster analyses. When alcohol consumption was reported, internal human factors (dizziness, erroneous actions) were commonly mentioned as a part of the factors involved in the accident mechanism, so some cases that reported no alcohol consumption, but similar “internal factors”, may have been attributable to alcohol. Therefore, a reduction in alcohol consumption could prevent a substantial number of fall injuries among adults.

Walking was the most common mechanism of preceding activity for fall injuries among the working and elderly age groups in Shenkursk District, as has been reported in the USA, the Netherlands, Japan, and India (Timsina et al. 2017; Dandona et al. 2010; Bleijlevens et al. 2010; Niino et al. 2000). Inside of a dwelling was the most common fall injury site among the elderly age group in our study, as well as in several studies from Australia and the USA (Peel et al. 2002; Timsina et al. 2017; Talbot et al. 2005). However, for example, in the Netherlands, the majority of falls among the elderly occurred outdoors (Bleijlevens et al. 2010). Our findings indicate that preventing falls among the elderly should primarily be done by addressing the safety of home and near-home environments (removal of slippery surfaces and items which may cause stumbling, installing guardrails, soft floorings) and measures to prevent weakness, dizziness, and loss of balance (proper medication, balance exercises, abstinence from alcohol).

### Strengths

This is, to our knowledge, the first Russian registry-based study investigating circumstances of fall injuries. The findings are similar to other studies, but since we failed to identify studies from Russia on this topic, our study adds new information in a cross-national perspective.

The present study was conducted using data from the population-based SHIR, an injury registry covering a defined geographical area. The data were collected using a standard IRF with a free-text field for recording a verbal description of how the injury occurred. These design features should have minimized selection and information biases in the description of the circumstances of fall injuries in the study area. This study also identified clusters of fall injuries in terms of preceding circumstances for each investigated age group, which were described as the most common fall injury circumstances (or “typical fall injury scenarios”), which represents a sensible evidence basis for planning age-specific preventive interventions.

### Limitations

The SHIR includes injuries which are treated at the Shenkursk CDH only. Based on our earlier study of completeness, representativeness, and reliability of the SHIR data (Unguryanu et al. 2019), 56% of all injuries in the district occur in rural areas, and 42% of the rural injuries are treated at primary care units. This means that about 20% of total injuries in the district are not treated in the CDH and, therefore, are not covered by the SHIR. Comparisons of rural injuries treated at the CDH to those treated at rural primary care units have shown that more severe cases are more commonly referred to the CDH than minor injuries. Thus, SHIR can be considered representative of all injuries in the district but with a consideration of the possible overrepresentation of severe injuries that occurred in rural areas. The same applies to falls addressed by this paper. However, as the proportion of injuries treated in rural primary health care units is limited to 20%, the bias is minor.

Another methodological consideration is completeness of the SHIR with respect to the coverage of cases treated at the CDH. According to our estimates (Unguryanu et al. 2019), this completeness was 86%. There were no substantial differences between registered and missed injuries in the SHIR by sex, weekday of admission, diagnostic and external cause categories, but missed cases had insubstantially higher proportions of child injuries and injuries in summer time (Unguryanu et al. 2019). However, as the proportion of missed injuries in the SHIR was small and the missed cases were similar to the registered ones, the imperfect completeness of the SHIR should not considerably affect its representativeness for total injuries as well as fall injuries treated at the CDH.

This study summarizes the fall injury panorama in the study area without considering sex differences in circumstances of fall injuries. We observed differences in selected characteristics of fall injuries between men and women, but they were assessed as indecisive for the planning of preventive measures and were therefore omitted.

We did not examine how circumstances of fall injuries in children and adults varied by socioeconomic status, as corresponding data were not available. Previous studies of falls have indicated that people with low socioeconomic status are at an increased risk of fall injuries and injuries in general (Wadhwaniya et al. 2017; Shenassa et al. 2004; Khambalia et al. 2006; Stewart et al. 2015). Logically, the effects of socioeconomic factors on the risk of falls are mediated by higher probabilities of exposures to unsafe environments and risky behaviors, like alcohol abuse and poor parental control. Such factors create fructuous contexts for injury circumstances, and the latter become the most proximate and modifiable factors in the causal chain. For that reason, they make up part of the focus of the data collection for the SHIR and our study.

We did not specifically address seasonal aspects of fall injuries. These require a separate investigation with a focus on fall injuries occurring outdoors. Finally, the results of the study have limited generalizability due to the fact that Shenkursk District is a relatively small area with a cold climate, largely rural characteristics, and a poorly developed infrastructure (low-rise buildings, largely unpaved roads, stove heating, and no tap water in many houses). On the other hand, our findings may be quite applicable in similar rural settings in the North of Russia, which may be considered deprived compared to urban settings.

## Conclusion

The circumstances of fall injuries in the study area varied across age groups. Most fall injuries in the preschool age group were due to climbing up on or down from home furnishings, while physical exercise with sport and play equipment were predominant fall circumstances among the school age group. Slipping on ice-covered surfaces was the most frequent fall injury circumstance in the working and elderly age groups. These findings can guide age-specific preventive strategies in the study area and similar settings.

## Data Availability

The anonymized datasets used and/or analysed during the current study are available from the corresponding author on reasonable request.

## Abbreviations

AIS: Abbreviated Injury Scale
ANOVA: Analysis of variance
CDH: Central district hospital
ICD: International Statistical Classification of Diseases and Related Health Problems
IRF: Injury registration form
SHIR: Shenkursk Injury Registry
